# An Easy and Reliable Method for Calibrating Combustion-Based Total Organic Carbon Analyzers for Solid Samples Containing Less than 100 μg of Carbon Using Polystyrene Microspheres

**DOI:** 10.1021/acs.analchem.6c00763

**Published:** 2026-08-31

**Authors:** Guillaume Bucher, Lina Büngener, Dora Mehn, Douglas Gilliland

**Affiliations:** 99013 European Commission, Joint Research Centre (JRC), Ispra 21027, Italy

## Abstract

Combustion-based total organic carbon (TOC) analyzers for solid samples are interesting tools for estimating the mass of microplastics in environmental samples. However, the low concentration of microplastics to be quantified may require TOC calibration below 100 μg of carbon (μgC), which could be laborious and tedious. This study proposes a fast, reliable, and easy-to-use calibration procedure that uses monomodal, compact polystyrene (PS) microspheres with nominal diameters of either 200 or 250 μm. Calibration curves obtained with the PS microspheres within the range of 8 to 50 μgC were linear and statistically equivalent to the reference calibration curve obtained with a diluted glucose solution. This demonstrates the effectiveness of this approach. This calibration procedure was successfully used to quantify the carbon content and accurately determine the size of unknown PS microspheres. The average size recovery was 98% ± 2% for 13 randomly selected particles ranging from 400 to 525 μm in diameter. Additionally, the study showed that the increase in carbon content following durable Nile Red staining of PS microspheres can be assessed using TOC analysis. This increase is likely due to organic solvent being trapped in the polymeric structure when PS particles are heated above their glass transition temperature. This alternative calibration procedure could improve the accuracy and ease of TOC analysis in various fields, including the monitoring of low microplastic content in environmental samples.

## Graphical abstract

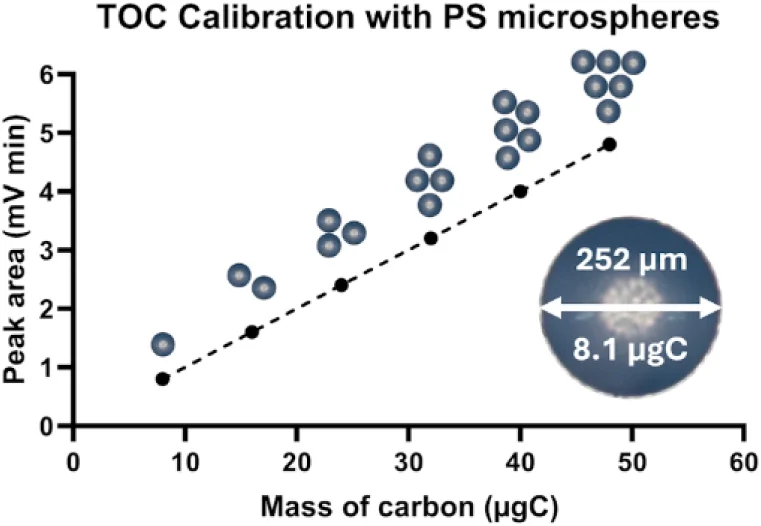

## Introduction

Up to the present day, the rather recent field of microplastic analysis struggles to find analytical methods that can identify and quantify plastic particles smaller than 1 mm with a sufficient level of specificity, while offering high sample throughput and not entailing excessive costs and analysis time. Spectromicroscopy methods, such as μFT-IR and μRaman, are able to identify polymer types down to a microplastic size of around 10 and 1 μm, respectively, while providing information on particle size and numbers, but analysis time per sample is often on the order of magnitude of days and instrument costs are comparatively high.^1,2^ Additionally, the conversion of number-based data to mass-based data requires assumptions about the three-dimensional characteristics of particles (e.g., thickness) and therefore involves a higher level of uncertainty. Thermo-analytical methods, such as Py-GC/MS and TED-GC/MS, allow the identification of polymer types and mass-based quantification of microplastics, but they are destructive and cannot provide any information on particle size, morphology, and number.

Total organic carbon (TOC) analyzers are universal carbon detectors, which is both their strength and their weakness. They detect and quantify any source of carbon present in a sample, including particulate and dissolved organic matter, carbonates, organic molecules (e.g., solvents, pesticides, pharmaceutical residues), and micro­(nano)­plastics. However, unlike Py-GC/MS, μFT-IR, and μRaman, TOC analyzers are not limited to detecting and quantifying a specific list of polymers. The latter are limited by nonexhaustive spectral databases, calibration issues, and spectral interferences such as fluorescence, particle thickness, and particle overlapping. If samples are “clean” enough, meaning they contain a low amount of particulate organic matter other than microplastics (e.g., pristine water or samples from remote areas), or if organic-rich samples undergo adequate treatment aiming to remove nonplastic organic matter (e.g., filtration, Fenton digestion, enzymatic digestion, and density separation), the residual measured TOC mass can be potentially attributable to microplastics and can serve as a powerful, inexpensive, and quick indicator of microplastic content.^3–12^ TOC can also serve as a tool for monitoring the efficiency of sample treatment (i.e., the removal of unwanted organic matter) and for estimating the optimal subsample volumes for polymer-specific mass-based techniques such as Py-GC/MS.^11^ Since microplastic mass concentrations are generally expected to be low in environmental samples, calibration below 100 μg of carbon (μgC) is important.

Most of the catalytic combustion-based TOC analyzers for solid samples with Non-Dispersive Infrared (NDIR) detection of CO_2_ claim a limit of detection (LOD) in the tens of μgC, typically from 15 to 100 μgC, with a maximum sample size ranging from 1 to 3 g of solid material. In 2021, Hong and coworkers demonstrated that it was possible to calibrate the SSM-5000A solid sample combustion unit (Shimadzu, Kyoto, Japan) for total carbon (TC), using a dilute solution of glucose, to reach an LOD of 3 μgC.^7^ Although very effective, this procedure remains laborious and requires a fair amount of time to accurately prepare, weigh, and dry various amounts of a dilute glucose solution in sampling boats to build a nice linear TC calibration curve below 100 μgC.

In this technical note, we propose an alternative, fast, reliable, and easy TC calibration procedure down to 8 μgC. This first-of-its-kind procedure uses monomodal, compact polystyrene (PS) microspheres with nominal diameters of either 200 or 250 μm. Additionally, we demonstrate how solid-based TOC analysis can be used to accurately estimate the diameter of individual microplastic beads and discuss the potential impact of in-house Nile Red staining on microplastic carbon content.

## Material and Methods

### Reagents and Materials

Ultrapure Milli-Q water (UPW) at 18.2 MΩ·cm was produced with a Millipore (Burlington, MA, USA) production system. Absolute ethanol, acetone, calcium chloride dihydrate (CaCl_2_·2H_2_O), Nile Red (C_20_H_18_N_2_O_2_, 75.45%C), and d-(+)-Glucose (C_6_H_12_O_6_, 40.00%C, 99.9% purity) were supplied by Sigma-Aldrich/Merck (Darmstadt, Germany). Polystyrene microspheres with nominal diameters of 200 μm and 250 μm were supplied as aqueous suspensions by Sigma-Aldrich/Merck (Darmstadt, Germany). Polystyrene beads from a 500–600 μm sieve fraction were supplied in dry form by Polysciences (Warrington, PA, USA). Table 1 summarizes the characteristics of the PS microparticles used in this study. TOC ceramic sample boats were supplied by Shimadzu (Kyoto, Japan) and analyzed in TC mode with a SSM-5000A solid sample combustion unit (900 °C, pure O_2_ carrier gas) to make sure they were clean and clear of any carbon-containing residue prior to use in this study.

**Table 1:** Characteristics of the PS Microparticles Used in This Study, Including Nominal Diameter, Diameter Reported on the Certificate of Analysis (CoA), and Estimated Carbon Content of a Single Particle

ID	Nominal diameter (μm)	Diameter on CoA (μm)	Carbon content^a^ (μg)
PS_200	200	204.0 ± 2.8 (1.3%)	4.306 ± 0.178 (4.1%)
PS_250	250	252.0 ± 6.8 (2.7%)	8.117 ± 0.657 (8.1%)
PS_SF2	500–600 sieve fraction	500–600 sieve fraction	63.403–109.561

a Considering the average diameter reported on the CoA, the PS density of 1.05 g cm^–3^ and 92.26%Carbon in PS.

### Durable Nile Red Staining

In order to make the PS_200 microspheres more visible and therefore easier to count and manipulate individually, durable Nile Red staining of PS_200 microspheres was adapted from the procedure described by Ponti et al. (2025).^13^ In brief, 100 μL of a 1 mg mL^–1^ Nile Red solution in acetone was added to approximately 100 mg of PS_200 particles suspended in 10 mL of an ethanol:water 1:1 mixture in a 35 mL microwave glass vessel. The particles were then stained using a Discover SP-D Microwave digestion system from CEM (Cologno al Serio, Italy) by applying 120 °C and 300 psi for 1 min, followed by filtration using a 10 μm Nylon filter from Millipore/Merck (Darmstadt, Germany) and final resuspension in ethanol.

### Glucose Reference Calibration

A dilute glucose standard solution at 4.015 μg μL^–1^ (equivalent to 1.606 μgC μL^–1^) was prepared gravimetrically by dissolving glucose powder into UPW. Then, TOC reference calibration standards were prepared by adding 5, 10, 20, 30, or 50 μL of the dilute glucose standard solution (corresponding to 8.030, 16.060, 32.119, 48.179, or 80.298 μgC) into clean TOC ceramic sample boats. The boats were dried for 1 h at 80 °C to evaporate the water prior to being analyzed in TC mode with a SSM-5000A solid sample combustion unit (900 °C, pure O_2_ carrier gas) coupled to a TOC-L CPH from Shimadzu (Kyoto, Japan) for carbon (as CO_2_) detection and quantitation. The CO_2_ concentration is plotted over time, typically resulting in one signal peak per sample. The glucose reference calibration curve was then constructed by plotting the signal peak area obtained via automated integration by the instrument software as a function of the carbon content for each calibration standard.

### PS Microspheres Calibration

PS microspheres (PS_200, PS_250, and PS_200_NR) were dispersed on a clean glass Petri dish using a small volume of ethanol (ca. 2–3 mL), which disperses the microspheres more effectively than water. This allowed the operator to pick (with a pipet) and transfer 1 to 12 individual PS microspheres into clean TOC ceramic sample boats. The Nile Red-stained microspheres (PS_200_NR) could be easily tracked using an Alonefire X901 UV 365 nm 10 W flashlight from Shenzhen Shiwang Lighting Co. (Shenzhen, China). The boats with deposited particles (prepared in triplicate) were dried for 1 h at 80 °C to completely evaporate the ethanol before being analyzed in TC mode using the SSM 5000A unit coupled to a TOC-L CPH. The PS microsphere calibration curves were then constructed by plotting the signal peak area obtained via automated integration by the instrument software as a function of the “total PS microspheres carbon content” calculated as the carbon content of one particle multiplied by the number of particles deposited in each sample boat.

### μRaman Analysis of PS Microspheres

PS microsphere images and Raman spectra from 150 cm^–1^ to 3200 cm^–1^ were acquired using a Renishaw inVia confocal microscope (Wotton-under-Edge, Gloucestershire, United Kingdom) to confirm the polymer’s identity and purity, and to observe any changes following Nile Red staining. Optical and Raman acquisitions were performed using a 10x objective lens and a 785 nm laser set at 100% power (100 mW), with an exposure time of 0.4 s and five replicate measurements.

### Empirical Estimation of PS Microsphere Densities

Seven solutions with densities ranging from 1.030 to 1.060 g cm^–3^ with 0.005 g cm^–3^ increments were produced by dissolving calcium chloride dihydrate in UPW in appropriate proportions. A mixture of PS_200 and PS_200_NR microspheres was suspended in 10 mL glass centrifuge tubes using 5 mL of each density solution. The seven resulting suspensions were then centrifuged at 20 °C for 5 min at 3000 rpm (1620 RCF) using an Eppendorf 5804R centrifuge (Hamburg, Germany) equipped with an A-4-44 swing bucket rotor. The position of the PS_200 and PS_200_NR microspheres (i.e., bottom or surface) was qualitatively determined by visual observation for each density solution and reported in Figure S1.

## Results and Discussion

### Calibration with Pristine PS Microspheres versus Glucose Reference

Using microplastic particles for the TOC calibration offers several advantages. First, this eliminates the need to prepare a freshly diluted standard solution of glucose. Thus, volumetric flasks, calibrated pipets, and an analytical balance are unnecessary. Ultimately, this reduces both the experimental error and labor time. However, calibrating with microplastic particles requires knowledge of the carbon content of a single particle with a sufficient level of precision. Therefore, it is highly advisable to verify the diameter of the microplastic particles claimed by the manufacturer on the CoA, evaluate the potential impact of additives present in the polymer (e.g., dye), and, if applicable, in the suspension (e.g., surfactant). All of these parameters may contribute to uncertainty when determining the carbon content. In this study, the average diameters of the PS_200 and PS_250 particles used for calibration were verified by optical microscopy and were within the uncertainty provided on the CoA (Table 1). The purity of the polymer constituting these particles in terms of the carbon content was not directly verified. However, Raman and Py-GC/MS analysis (data not shown) confirmed the identity of the polymer (i.e., PS) with traces of styrene monomer. The potential presence of additives (e.g., surfactant) in the commercial PS microsphere suspensions was considered as being negligible after the particles (i.e., a few microliters of a 10% wt. suspension) were dispersed in a comparatively large (i.e., 2–3 mL) amount of ethanol to be picked individually with a pipet.

Calibration curves obtained for PS_200 and PS_250 microspheres (Figure 1) were linear (all *R* ^2^ > 0.995) and statistically equivalent to the reference calibration curve obtained with glucose (*P*-value > 0.05 with GraphPad Prism software version 10.1.2 slope and intercept comparison module).^14^ In practice, PS_250 microspheres were much easier to manipulate and count once transferred into the sample boats. The instrument was systematically able to detect and quantify a single PS_250 microsphere (8.117 μgC), but it was not able to detect a single PS_200 microsphere (4.306 μgC). This result was in line with the limit of quantitation (LOQ) of our instrument in TC mode, which was empirically determined at 8 μgC with the diluted glucose standard solution. It should be noted that even though the quantitation of two PS_200 microspheres (2 × 4.306 = 8.612 μgC) was possible, the software occasionally failed to detect and quantify the signal peak. Unfortunately, the software automatically performed peak detection and integration and could not be forced manually. Consequently, peaks with an area smaller than 1 mV min, as it was the case at the LOQ level, were very small and were sometimes not detected by the software. Therefore, it was decided to start the calibration for PS_200 with three microspheres, which the software systematically detected and quantified.

**Figure 1:**
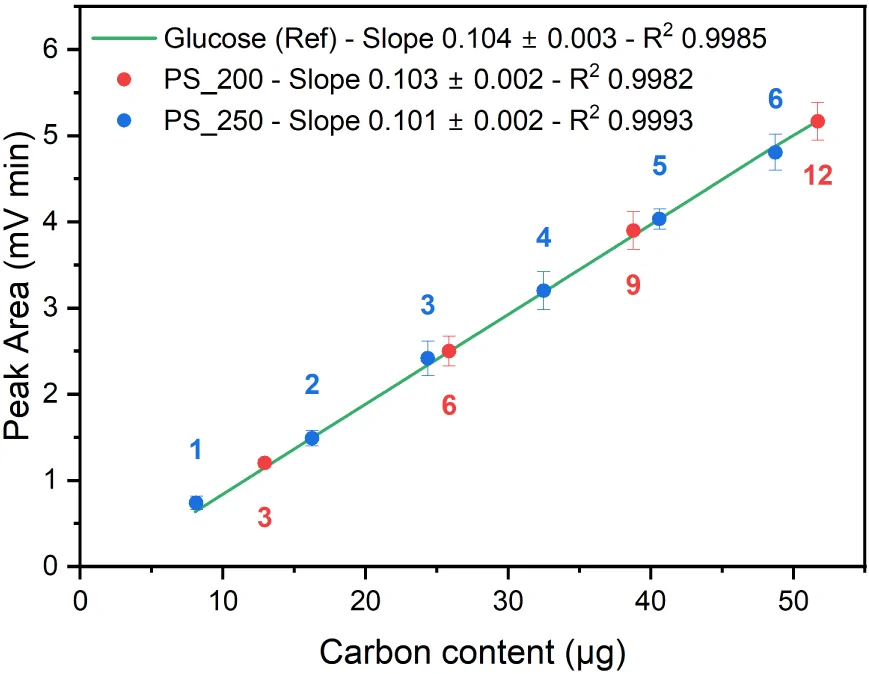
Reference organic carbon calibration curve obtained using a glucose solution (green solid line) in the range of 8 to 50 μgC, compared to the calibration points obtained with PS_200 (red dots) and PS_250 (blue dots) microspheres. Linear regression statistics (Slope ± SD and *R* ^2^) are included in the figure for each calibration. The number of particles placed in each sample boat is indicated by the number next to each data point.

### Impact of Durable Nile Red Staining on Individual Particle Carbon Content

PS_250 microspheres were easy to visualize, count, and manipulate with the naked eye. This was slightly more challenging with PS_200 microspheres, though still possible depending on the operator’s skills. Therefore, it was decided to durably stain the PS_200 microspheres with Nile Red to make them more visible under UV light (365 nm) and easier to manipulate. The calibration curve obtained with PS_200_NR and presented in Figure 2 (light purple dots and dashed line) was linear (*R* ^2^ = 0.9995) but showed a statistically significant increase in slope compared to PS_200 (*P*-value < 0.05 with GraphPad Prism software version 10.1.2 slope and intercept comparison module).^14^ This meant that the staining process somehow increased the carbon content of the PS_200_NR microspheres compared to the pristine PS_200 microspheres. Consequently, the theoretical carbon content of PS_200 microspheres (see Table 1) could no longer be used when calibrating with PS_200_NR microspheres. However, the linearity of the calibration curve obtained with PS_200_NR microspheres and the similar variability in terms of peak area compared to PS_200 microspheres suggested that the carbon content increase was quite homogeneous among the microspheres. The average excess of area observed per individual PS_200_NR particle was converted to the equivalent mass of carbon in excess per particle (*m*_NR_) using the glucose reference calibration curve. The value of 0.19 ± 0.09 μgC was determined for *m*_NR_, meaning that PS_200_NR microspheres would contain on average 4.496 μgC, which is 4.4% more carbon than pristine PS_200 microspheres.

**Figure 2:**
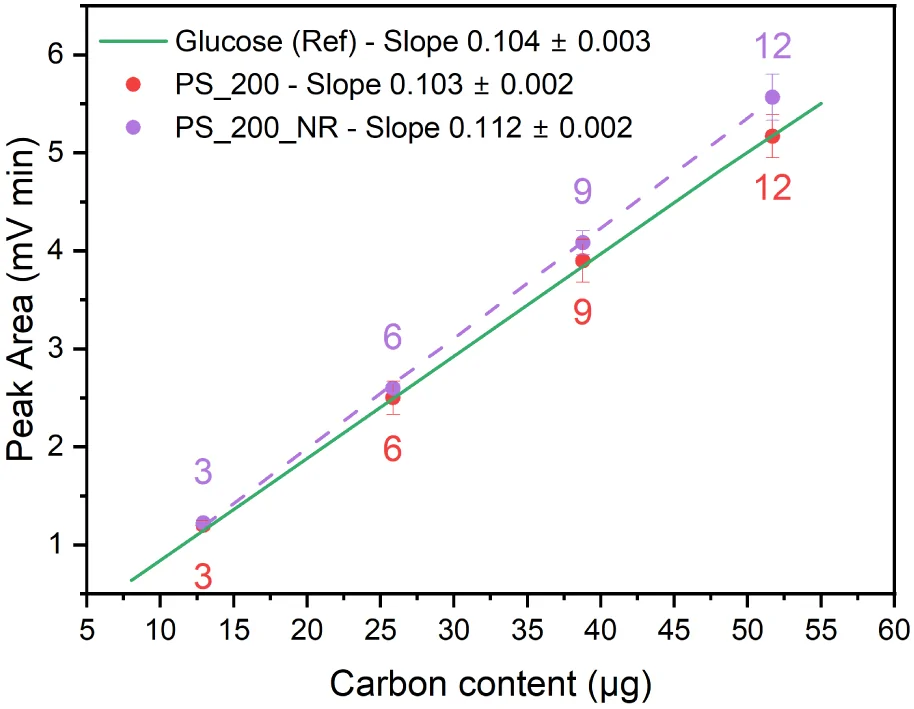
Calibration curves obtained with a glucose solution (Reference, green solid line), with unstained PS_200 (red dots), and with Nile Red-stained PS_200_NR (purple dots and dashed line). Linear regression statistics (Slope ± SD) are included in the figure for each calibration. The number of particles placed in each sample boat is indicated by the number next to each data point.

Can Nile Red incorporation account for the increased carbon content? Taking into consideration the quantities of PS_200 microspheres (100 mg, equivalent to about 21416 particles) and Nile Red (100 μg) introduced for the staining process, the maximum amount of Nile Red incorporated in each PS_200_NR microsphere would be on average 0.0047 μg, which corresponds to about 0.0035 μgC. This amount of carbon represents less than 0.1% of the PS_200 microsphere carbon content and about 1.8% of the estimated *m*_NR_ value. Nile Red should therefore not significantly impact the carbon content of PS_200_NR microspheres.

What can then explain *m*_NR_? The Raman spectra of PS_200, PS_250, and PS_200_NR (Figure S2) were all comparable and consistent with polystyrene.^15^ Only one minor peak at 1631 cm^–1^ corresponding to the vinyl bond stretch of styrene monomer became missing in the PS_200_NR spectra after the staining process, suggesting the potential leaching of residual monomers initially trapped inside the PS particles.^16,17^ Additionally, following imaging of the microspheres using the μRaman’s microscope, no significant changes in shape but a slight increase in diameter (+2% diameter equivalent to +6.2% volume) was observed between PS_200 (204.0 ± 2.8 μm) and PS_200_NR (208.1 ± 2.5 μm). This variation of particle diameter was accompanied by a change in particle density. The density of the PS_200_NR microspheres was estimated empirically to be between 1.030 and 1.035 g cm^–3^, while the density of PS_200 was confirmed to range between 1.045 and 1.050 g cm^–3^ as expected for PS (Figure S1). The trapping of ethanol (0.789 g cm^–3^) during the staining process, which could occur above the glass transition temperature of PS (*T*_gt_ ≈ 100 °C), may potentially explain the lower density of PS_200_NR microspheres compared to pristine PS_200.^18^ In fact, if the extra volume were filled with ethanol, the PS_200_NR microspheres would have a theoretical density of 1.035 g cm^–3^ and a carbon content of 4.419 μgC. These theoretical values are consistent with the estimated density and measured carbon content of PS_200_NR. In addition, the PS_200_NR microspheres appeared visually opaque compared to the transparent pristine PS_200. This could also be another indication that ethanol was trapped inside the polymeric structure. It should be noted that the PS_200_NR particles were heated above *T*_gt_ for 2 h in an attempt to reverse the potential trapping of ethanol. However, this was unsuccessful, and the carbon content remained unchanged.

Consequently, on the basis of a body of circumstantial evidence, the increased carbon content observed for PS_200_NR microspheres was likely due to the incorporation of both Nile Red and other carbon-based species such as ethanol, which was present in large excess (5 mL) during the staining process. These species can enter the polymeric structure when they are heated above the glass transition temperature (*T*_gt_) and become trapped during cooling.

### Microsphere Sizing Using TOC

Thirteen individual PS microspheres randomly selected from a sieve fraction of nominal size ranging from 500 to 600 μm were sized using the μRaman microscope and individually analyzed using the TOC. The mass of carbon obtained from TOC using the PS_250 calibration for each microsphere was then converted to an equivalent spherical diameter using the assumption of perfect PS spheres with a density of 1.05 g cm^–3^ and a carbon fraction of 92.26%. Figure 3 presents the particle diameter obtained from TOC measurement as a function of the actual diameter measured with the μRaman microscope. Both diameters were highly correlated with Pearson’s correlation coefficient (*r*) equal to 0.9999 and a slope of 0.979 ± 0.005. In other words, the diameters obtained by converting the TOC measurements into particle diameters were in agreement with the microscope measurements. The average size recovery (defined as TOC diameter/Microscope diameter) for the 13 randomly selected particles was 98% ± 2% (*n* = 13), in-line with the observed slight underestimation of the diameter obtained from TOC (slope < 1).

**Figure 3:**
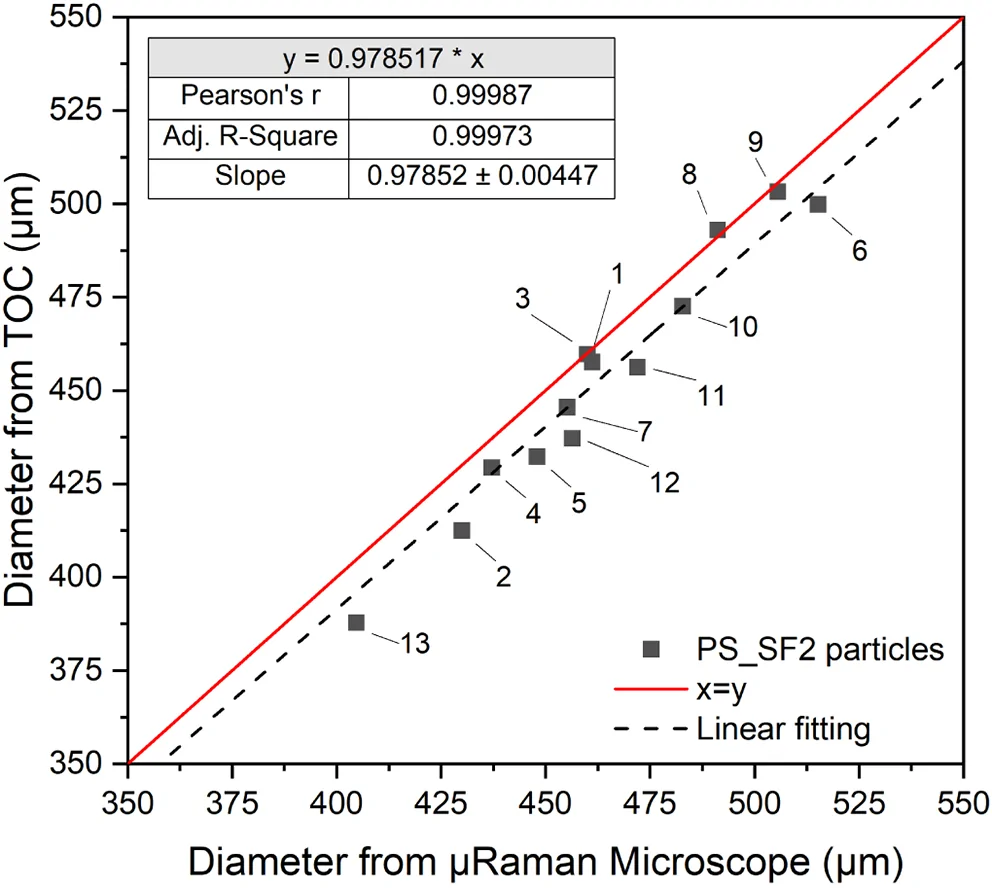
Diameter obtained for 13 individual PS particles from TOC measurement as a function of the actual diameter measured with the μRaman microscope. The red solid line represents *x* = *y*, and the black dashed line represents the linear fitting from the 13 data points with statistics detailed in the table insert.

It is important to note that the TOC approach for sizing single microplastic beads is not limited to polystyrene; it can be applied to any carbon-containing polymer. The main limitation stems from the assumption that the particles are spherical. Then, depending on the polymer’s characteristics (i.e., polymer density and percentage of carbon in the polymer chain), the smallest spherical particle that can be detected as a single particle considering a LOQ at 8 μgC (see eq S1) would have a diameter ranging from 250 μm (e.g., PS, PET, and PC) to 310 μm (e.g., PTFE and PVC). With a little practice, particles of that size range are in fact easy to visualize, manipulate, transfer, and count one-by-one using a pipet once dispersed in a small volume of adequate solvent in a Petri dish.

## Conclusion

The proposed alternative calibration procedure using monomodal, compact, PS microspheres of 200 or 250 μm nominal diameters was shown to be fast, reliable, and easy to use for total carbon (TC) calibration down to 8 μgC. The calibration curves obtained for PS_200 and PS_250 microspheres in the sub-100 μgC range were linear and statistically equivalent to the reference calibration curve obtained with glucose, demonstrating the effectiveness of this method. The calibration obtained with the proposed procedure can therefore be used for any kind of TOC analysis in the low concentration range. The durable Nile Red staining procedure was found to significantly increase the carbon content of PS_200 microspheres, likely due to the trapping of ethanol into the polymeric structure when PS particles are heated above the glass transition temperature. Consequently, despite the ease of manipulating stained PS microspheres, it would not be recommended to use them for calibration purposes, unless their density and carbon content are precisely known. Additionally, since TOC calibrations are often used for several months, it is essential to analyze control samples regularly, ideally each time the instrument is used. Using microplastic particles for this purpose is also fast and convenient. The analyst would simply transfer a restricted number of microplastic beads with a known carbon content into a TOC sample boat, analyze the sample, and check the carbon content recovery. This control strategy using microplastic particles can be applied whether the calibration was performed with microplastic particles or with a diluted glucose solution. Finally, the study demonstrated that, with a LOQ at 8 μgC, TOC can be used to accurately estimate the diameter of individual spherical microplastic particles with diameters above 250 to 310 μm depending on the polymer type, with a high correlation between TOC measurements and microscope measurements.

## Supplementary Material



## Notes

μRaman analysis of PS_200, PS_200_NR, and PS_250; equation for the estimation of LOD_size_ and empirical estimation of PS_200 and PS_200_NR microspheres densities (PDF)

## Abbreviations

TOC: total organic carbon
TC: total carbon
NDIR: nondispersive infrared (detector)
PS: polystyrene
μgC: μg of carbon
