# Supplementary material for: An Easy and Reliable Method for Calibrating Combustion-Based Total Organic Carbon Analyzers for Solid Samples Containing Less than 100 μg of Carbon Using Polystyrene Microspheres
Source: Anal Chem. 2026 Aug 31;98(36):26284–9. doi: 10.1021/acs.analchem.6c00763 (PMC13584624; doi:10.1021/acs.analchem.6c00763)
Supplement: Supplementary file 1 [file ac6c00763_si_001.pdf]

An easy and reliable method for calibrating combustion-based total organic carbon analyzers for solid samples containing less than 100 µg of carbon using polystyrene microspheres

Guillaume Bucher[1], Lina Büngener[1], Dora Mehn[1] and Douglas Gilliland[1]*

[1]European Commission, Joint Research Centre (JRC), 21027 Ispra, Italy.

Guillaume.bucher@ec.europa.eu; Lina.buengener@ec.europa.eu; *Dora.mehn@ec.europa.eu;
Douglas.gilliland@ec.europa.eu

Supporting Information

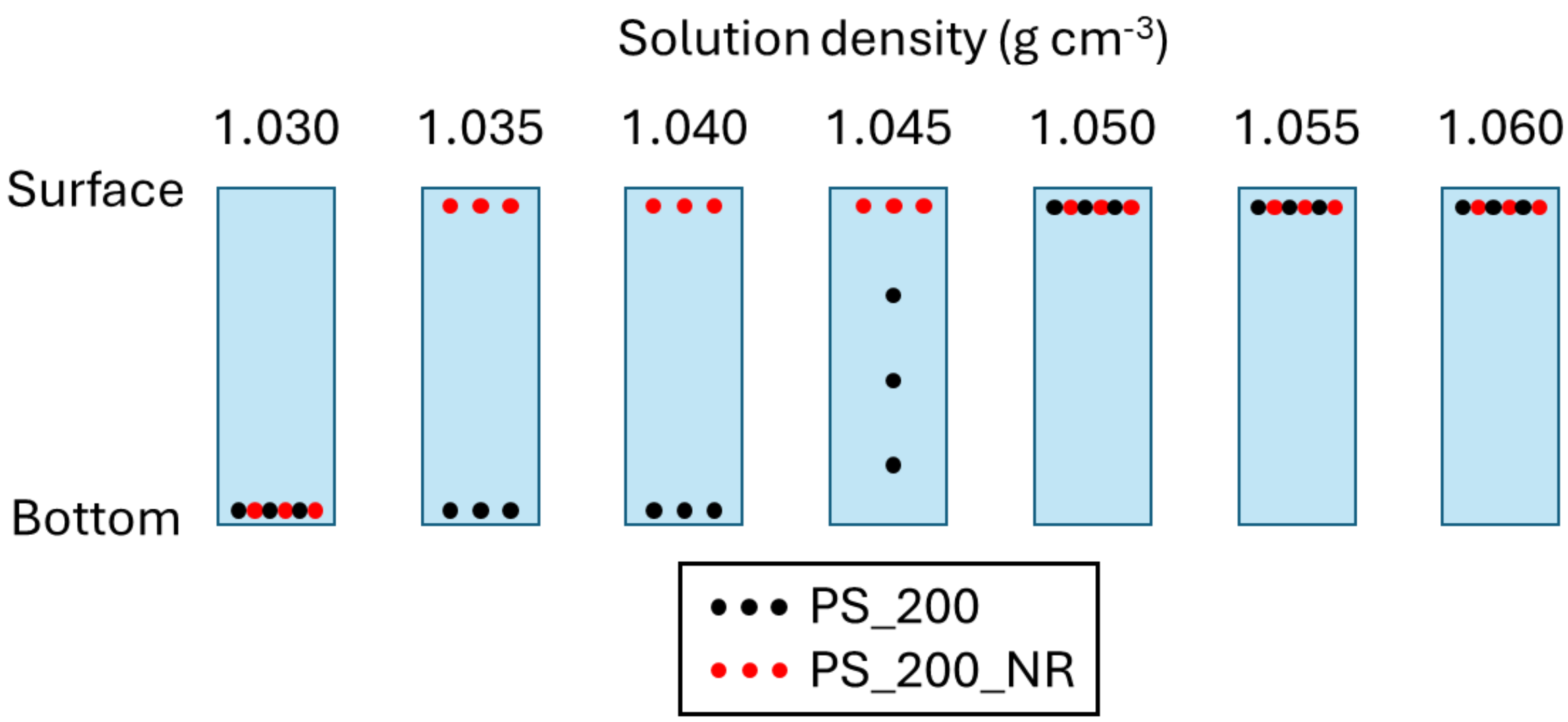

Figure S1. Schematic representation of the observed results from the empirical estimation of PS microspheres densities: A mixture of PS_200 (black dots) and PS_200_NR (red dots) microspheres was suspended in a series of seven $CaCl_2$ solutions with densities ranging from 1.030 to 1.060 g cm^{-3}. The seven resulting suspensions were centrifuged at 20 °C for 5 min at 1620 RCF. The final position of the PS_200 and PS_200_NR microspheres (i.e. bottom, middle or surface) was qualitatively determined by visual observation for each density solution and schematically reported in Figure S1. The density of the microspheres was empirically estimated as the density of the solution at which the particles began to float. For PS_200_NR, this was between 1.030 and 1.035 g cm^{-3}, and for PS_200, it was between 1.045 and 1.050 g cm^{-3}.

Equation S1. The diameter of the smallest spherical polymeric particle that can be detected as a single particle by the TOC is described as LOD_{size} and can be estimated using the following equation:

$$LOD_{size} = \left(\frac{6 \times m_c}{\rho \times \pi \times \%C}\right)^{1/3} \times 100$$

Where:

- LOD_{size} is the diameter (µm) of the smallest spherical polymeric particle that can be detected as a single particle by the TOC instrument
- m_c is the LOQ of the TOC instrument in µg of carbon (i.e. 8 µg in this study)
- ρ is the density of the polymer in g cm^{-3} (e.g. 1.05 g cm^{-3} for PS)
- $\%C$ is the percentage of carbon in the polymer chain (e.g. 92.26% or 0.9226 for PS)
- Correction factor (× 100) converts LOD_{size} in µm

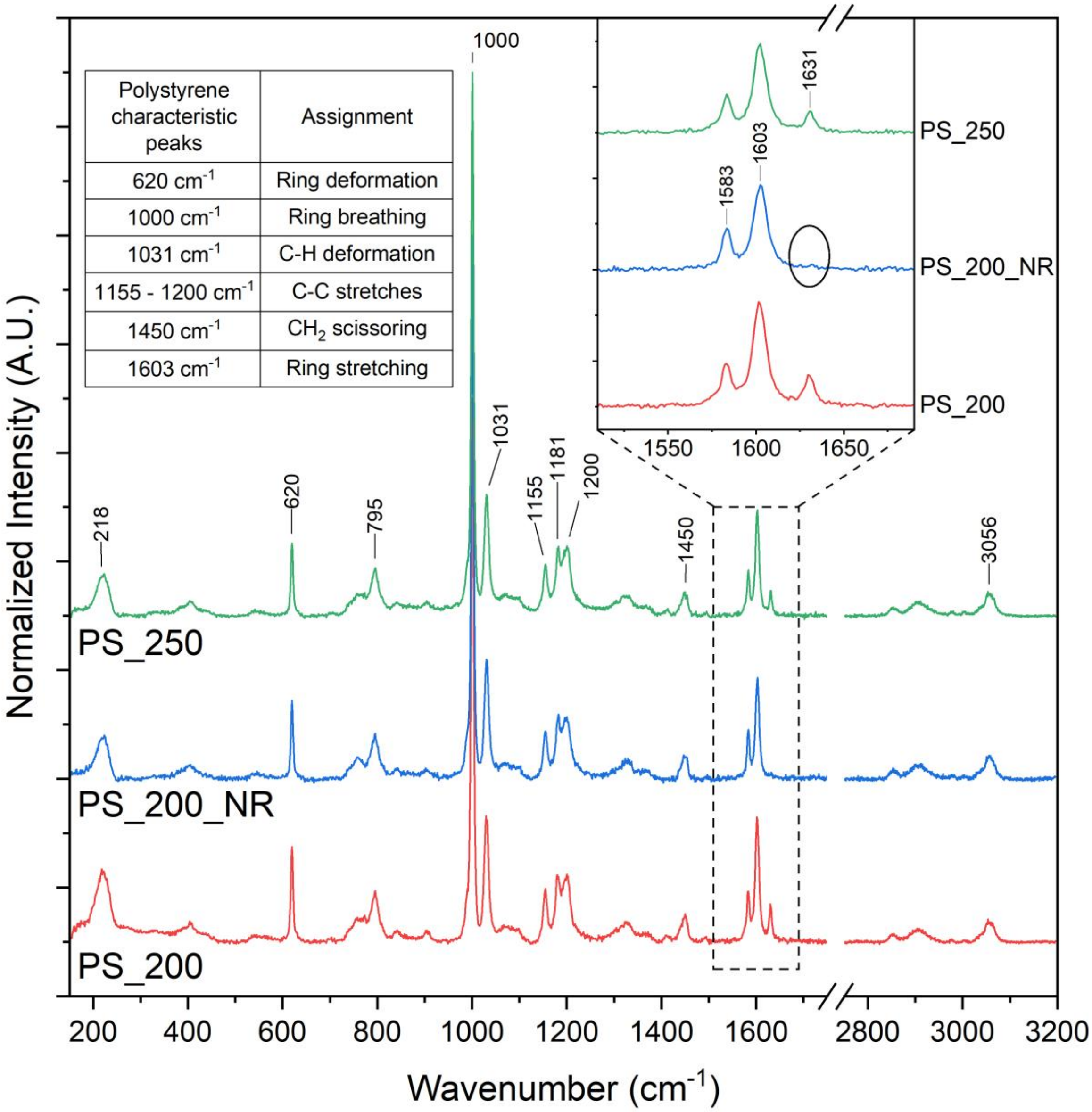

Figure S2. Raman spectra of PS_200 (bottom, red line), PS_200_NR (middle, blue line) and PS_250 (top, green line) particles. Intensity normalized versus the highest peak at 1000 cm^{-1}. The assignment of polystyrene characteristic Raman peaks is reported from Bridges, *et al.* (2024).[1]

REFERENCES

(1) Bridges, T. E.; Houlne, M. P.; Harris, J. M. Spatially Resolved Analysis of Small Particles by Confocal Raman Microscopy: Depth Profiling and Optical Trapping. *Anal. Chem.* **2004**, *76* (3), 576–584. https://doi.org/10.1021/ac034969s.
